# Jaguar Density at the Northeastern Limit of Its Distribution in Mexico

**DOI:** 10.1002/ece3.72932

**Published:** 2026-02-03

**Authors:** Zavdiel A. Manuel‐de la Rosa, Leroy Soria‐Díaz, Carlos Barriga‐Vallejo, Gabriela R. Mendoza‐Gutiérrez, Nayeli Martínez‐González, Claudia C. Astudillo‐Sánchez, José Jiménez

**Affiliations:** ^1^ Instituto de Ecología Aplicada Universidad Autónoma de Tamaulipas Ciudad Victoria México; ^2^ Facultad de Medicina Veterinaria y Zootecnia “Dr. Norberto Treviño Zapata” Universidad Autónoma de Tamaulipas Ciudad Victoria México; ^3^ Pronatura Noreste Monterrey México; ^4^ Facultad de Ingeniería y Ciencias Universidad Autónoma de Tamaulipas, Centro Universitario Victoria Ciudad Victoria México; ^5^ Instituto de Investigación en Recursos Cinegéticos (IREC, CSIC‐UCLM‐JCCM) Ciudad Real España

**Keywords:** camera trapping, density, jaguar, northeastern range limit, population size, random thinning spatial capture–recapture

## Abstract

Reliable estimates of population density are essential for the conservation of apex predators such as the jaguar ( *Panthera onca* ), particularly in peripheral regions of their distribution where existing data are insufficient to guide effective management. In Mexico, northeastern landscapes remain underrepresented in jaguar research, limiting the development of context‐specific conservation strategies. To address this gap, we conducted a camera trap survey in the El Cielo–Sierra de Tamalave biological corridor, a transitional zone located at the northeasternmost limit of the species' range. Over a 91‐day sampling period, we deployed 104 cameras across 52 paired stations and applied a random thinning spatial capture–recapture model (rt‐SCR), which integrates both identified and unidentified photographic detections. This represents the first application of rt‐SCR to jaguar data in Mexico. The model yielded a density estimate of 1.29 (0.93–1.70) individuals per 100 km^2^, with adequate goodness‐of‐fit across multiple detection metrics. Despite low detection rates, the rt‐SCR framework allowed for robust inference by maximizing data use and mitigates the loss of precision associated with excluding unidentified detections. Our findings provide a baseline for future monitoring in northeastern Mexico and demonstrate the utility of rt‐SCR models in data‐limited contexts. These results support the implementation of localized conservation actions and long‐term monitoring programs in peripheral jaguar habitats, where population viability may depend on maintaining ecological continuity and minimizing anthropogenic pressures.

## Graphical abstract

This study presents the first estimate of jaguar density in northeastern Mexico using a spatial capture–recapture model with random thinning, which incorporates both identified and unidentified camera trap detections. Conducted over 91 days with 104 cameras, the model yielded a density of 1.29 individuals per 100 km^2^ in the El Cielo–Sierra de Tamalave corridor. The results provide a baseline for monitoring and support conservation efforts in peripheral habitats with limited data availability.

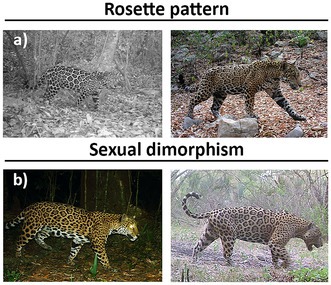

## 1 Introduction

The jaguar ( *Panthera onca* ) plays a critical role in Neotropical ecosystems as an apex predator and umbrella species (Ceballos et al. 2016). It also holds deep cultural significance in Mesoamerica (Sunquist and Sunquist 2002). Despite its ecological and symbolic importance, the size of the jaguar population in Mexico has remained poorly documented due to limited field research (Figel et al. 2022). In particular, systematic ecological studies are lacking in northeastern Mexico, which lies near the northeastern limit of the species' range, unlike the central and southern regions that have traditionally received more attention in conservation and monitoring initiatives (Charre‐Medellín et al. 2021; Amador‐Alcalá et al. 2024). This limited spatial representativeness reduces the ability to assess population patterns at a national scale and hinders decision‐making for the species' conservation.

Jaguars typically occur at low densities and depend on large, well‐connected habitats to fulfill their ecological requirements (Ceballos et al. 2016; Quigley et al. 2017). Their territorial behavior makes them particularly susceptible to habitat fragmentation and human disturbance (Tobler and Powell 2013; Gil‐Sánchez et al. 2021). In northeastern Mexico, habitat loss and fragmentation—mainly due to livestock expansion and infrastructure development near protected zones—pose significant threats to jaguar persistence by reducing available habitat, disrupting the function of biological corridors (Rosas‐Rosas et al. 2020; Thornton et al. 2020; Amador‐Alcalá et al. 2024) and increasing human–wildlife conflict (Luja et al. 2022). Critically, the absence of reliable population data in these changing landscapes limits the ability to assess conservation risks and hinders the implementation of evidence‐based management strategies (Ceballos et al. 2021).

In response to these conservation challenges, non‐invasive techniques—particularly camera trapping—have become essential tools for studying jaguar ecology. These devices allow researchers to estimate key ecological parameters such as population size, density, activity patterns, and behavioral responses to human pressures or interactions with other species (Anile et al. 2021; Mooring and Eppert 2022). Among these, population density stands out as a central metric in wildlife management, as it informs conservation planning by quantifying the number of individuals within a given area (Jiménez et al. 2023). Collaborative efforts involving civil society and governmental organizations—such as Alianza Jaguar, Panthera, and the U.S. Fish and Wildlife Service (USFWS)—have supported monitoring initiatives aimed at generating reliable data for long‐term conservation (Williams et al. 2002; SEMARNAT 2010; Quigley et al. 2017; Sanderson et al. 2022). However, jaguar density estimates across Mexico show considerable variation, ranging from 6.1 individuals/100 km^2^ in core habitats to 0.3 individuals/100 km^2^ in peripheral regions (Ceballos et al. 2016; Amador‐Alcalá et al. 2024), underscoring the need for context‐specific assessments.

Estimating population density is inherently complex because observed variation can arise not only from true differences in abundance but also from heterogeneity in both abundance and detection. Such heterogeneity may be driven by factors, including habitat characteristics, prey availability, and sampling constraints. Detectability, in particular, can vary with human activity or be limited by topographical and logistical challenges (Charre‐Medellín et al. 2021; Hidalgo‐Mihart et al. 2025). This complexity is further compounded by interactions with other species; for jaguars, these include apex predators, such as the black bear ( *Ursus americanus* ) and the cougar ( *Puma concolor* ), whose presence can lead to interspecific competition or niche partitioning (Contreras‐Díaz et al. 2021), ultimately influencing both abundance and detection probability at local scales.

Hierarchical frameworks such as spatial capture–recapture (SCR) have greatly advanced density estimation by explicitly modeling detectability, reducing biases associated with heterogeneity and enabling formal goodness‐of‐fit (GoF) testing (Efford 2004; Royle et al. 2014). However, a persistent challenge in SCR studies is the exclusion of photographic records lacking individual identification—typically due to partial views, blurred images, or single‐flank captures. These omissions reduce sample size and compromise precision estimates, particularly in low‐density contexts, such as peripheral areas of a species' range. To overcome this limitation, the random thinning SCR model (rt‐SCR) was developed as an extension of the SCR framework that incorporates unidentified detections through a probabilistic thinning process, retaining the mechanistic structure of SCR while maximizing data use (Anile et al. 2024). This approach could be especially relevant in range‐edge regions, where abundance and detection rates are expected to be low—a pattern consistent with the abundant‐center and rare‐edge hypotheses (Sexton 2024).

Because peripheral regions are characterized by low abundance and limited detections, we tested whether rt‐SCR improves the reliability of density estimates under these conditions. Northeastern Mexico represents such a peripheral region and is strategically important for jaguar conservation due to its exceptional ecological diversity and the convergence of Nearctic and Neotropical biotas (Steinberg et al. 2014). This area includes key mountain ranges, such as the Sierra de Tamalave, recently designated as part of the Monarch Butterfly Natural Landscape (Gobierno del Estado de Tamaulipas et al. 2022). These ranges maintain connectivity between jaguar populations, linking habitats from Cumbres de Monterrey National Park to the Abra Tanchipa Biosphere Reserve (Carrera‐Treviño et al. 2016), and form the El Cielo–Sierra de Tamalave biological corridor (Gómez et al. 2018). Assessing jaguar presence and density in this region is essential for evaluating the effectiveness of current protection measures and identifying critical gaps in conservation planning.

To address the challenges posed by low detection rates and partial individual identification, our goal was to estimate jaguar population density (*D*) and population size (*N*) across the state space of the El Cielo–Sierra de Tamalave biological corridor, located at the northeastern limit of the species' range. To maximize data use in this data‐limited context, we applied the random thinning extension of the spatial capture–recapture (rt‐SCR) framework, which mitigates the loss of precision associated with unidentified photographic records. By generating robust density estimates in a transitional and understudied region, our study provides critical insights into jaguar ecology and informs adaptive conservation strategies aimed at maintaining connectivity and long‐term population viability in northeastern Mexico.

## 2 Materials and Methods

### 2.1 Study Area

**FIGURE 1:**
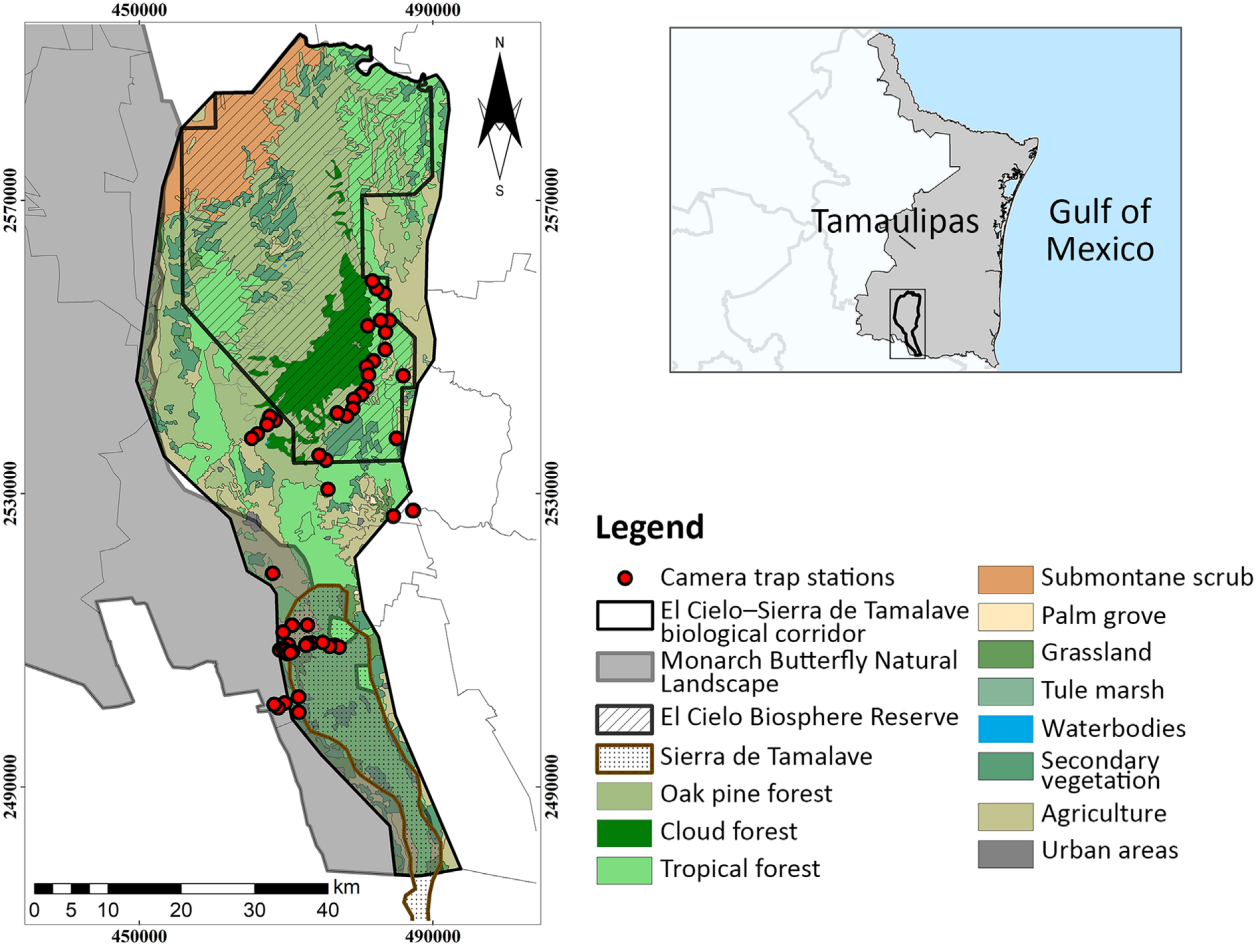
Spatial delimitation and geographic context of the El Cielo–Sierra de Tamalave biological corridor, with red points representing the “detectors” used for the spatial capture–recapture model.

The El Cielo–Sierra de Tamalave biological corridor is located in the southwest of the state of Tamaulipas, Mexico, and covers an area of 2799 km^2^ (Figure 1). The site's topography is influenced by the presence of the Sierra Madre Oriental and its branches. In the area, there are two protected sites due to the wide variety of ecoregions with Neotropical and Neartic species associated with a relatively small geographic area (Steinberg et al. 2014; Gómez et al. 2018). In the northern portion lies the El Cielo Biosphere Reserve, covering a total of 1445.30 km^2^, which has been under protection by the government of Tamaulipas since 1985 (Sosa et al. 1997). In the southwest lies the Sierra de Tamalave, which was designated in 2022 as part of the Protected Natural Area Monarch Butterfly Natural Landscape by the state government. This protected area covers 6.93% of Tamaulipas's total surface area (Gobierno del Estado de Tamaulipas et al. 2022). In addition to jaguars, these protected areas are host populations of several threatened carnivores, such as the black bear, ocelot ( *Leopardus pardalis* ), and margay ( *L. wiedii* ) (SEMARNAT 2010). The combination of abiotic factors such as altitude, latitude, proximity to the sea, and topography results in a variety of climates. In the lowland areas, the climate is warm and subhumid, with a dry season from November to March (Carrera‐Treviño et al. 2016). At altitudes above 800 m above sea level, a warm, humid, and temperate climate prevails, with 7 months of rainfall (April to October). The annual mean temperature ranges between 14°C and 25.5°C (Medrano 2005; Morrone 2005).

Within the monitoring site, forest stands provide suitable habitat for jaguars. However, structural connectivity across the landscape is compromised because the area is bordered by disturbed zones resulting from anthropogenic activities, primarily agricultural expansion and road construction. These alterations particularly affect low‐slope areas and the rolling hills that intersect and delimit the mountainous region, thereby reducing connectivity between the Sierra de Tamalave and the El Cielo Biosphere Reserve. Consequently, jaguars are forced to move primarily through mountainous sectors that retain primary and secondary vegetation (Gómez et al. 2018).

### 2.2 Sample Collection

The jaguar population was monitored using 104 infrared‐triggered camera traps (Cuddeback H‐1453, ScoutGuard SG565, StealthCam QS, USA) arranged in 52 paired stations (with one camera facing another), the Cuddeback H‐1453, ScoutGuard SG565, and StealthCam QS units have comparable detection capabilities, with trigger speeds ranging from 0.2 to 0.5 s, infrared no‐glow or low‐glow flash, detection ranges of 15–25 m, and detection angles between 40° and 55°. These specifications are adequate for detecting medium‐ and large‐sized mammals in rugged terrain. All models capture still images of at least 12 megapixels and operate with passive infrared sensors suitable for the photo‐identification of individually marked felids, which allowed photographs of both sides of the individuals, facilitating individual identification (Alves et al. 2024). The monitoring covered 91 days, from 15 February 2023 to 16 May 2023 (Figure S1). The traps were deployed into two areas (Figure 1); in the north, the minimum convex polygon covered 33,356 ha, and in the south, 6592 ha.

**FIGURE 2:**
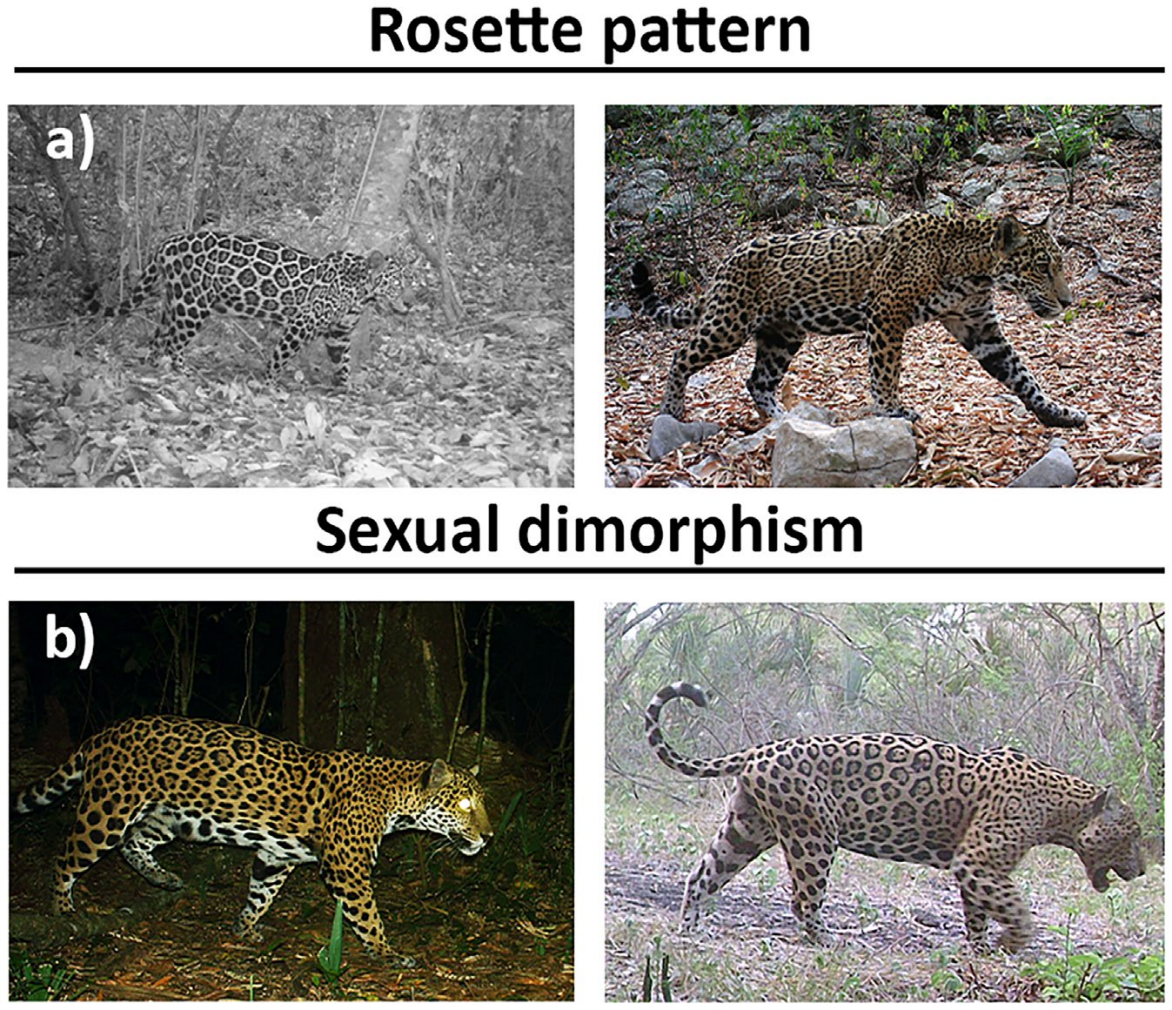
Identification of individual jaguars using camera traps combines (a) unique rosette patterns for each individual, (b) morphological differences between males and females, such as head size and the presence of a testicular sac. Image credit: Carlos Barriga‐Vallejo/Pronatura Noreste.

We selected camera trap locations based on direct or indirect evidence of fauna presence (scats, tracks, scrapes, and trails) to increase detection probability (Medellín et al. 2006). Camera traps were installed at a height of 60 cm above ground level without any bait, on rock walls and trees along natural trails, with an average nearest neighbor of 1308 m (range: 103–7196.7 m) between them. This spacing was selected to maximize the probability of detecting the same individual at adjacent stations, based on expected movement ranges of jaguars and previous studies using SCR models (e.g., Tobler and Powell 2013; Royle et al. 2014). Camera traps operated continuously (24 h) and were set to take a sequence of two images per activation with medium sensitivity, reporting date and time stamp, with a delay interval of 1.5 s between events. The identification of individual jaguars from camera trap images was achieved using morphological and phenotypic traits, such as the rosette pattern, which is unique to each individual (Aranda 2012; Alves et al. 2024). Sex identification was based on the presence of the scrotal sac in males and its absence in females (Figure 2) (Karanth and Nichols 1998). For reference, each jaguar was assigned a unique alphanumeric identifier (ID), consisting of the initial of the species' common name (J), sex (M‐F‐N), and an individual number. For the processing and management of photo captures, the DigiKam software was used, allowing the addition of tags to the metadata of each photograph, thereby facilitating organization and subsequent analysis (Pérez‐Solano 2019).

The camtrapR package (Niedballa et al. 2016) was used to create a database containing the metadata of each photograph. This database included individual IDs (when identifiable), date and time of capture, and the identifier of the station where the capture occurred. This information enabled the construction of three‐dimensional matrices for identified photographs (individual × trap × occasion) and two‐dimensional matrices for unidentified photographs (trap × occasion).

### 2.3 Population Density Estimate

A common issue when estimating densities from camera trap data is the exclusion of certain photographic records due to the inability to identify individuals. This typically results from partial views, blurred images, or photographs capturing only one flank. Such exclusions reduce sample size and compromise the reliability of density estimates. Although specific SCR approaches have been developed to handle incomplete bilateral information (Augustine et al. 2018), we addressed this problem using the random thinning model (Jiménez et al. 2021). This approach retains the mechanistic structure of SCR while incorporating unidentified detections, enabling the use of all camera trap data—including those usually discarded in regular SCR models (Anile et al. 2024; Hidalgo‐Mihart et al. 2025). A conceptual diagram of the rt‐SCR framework, including the thinning process and integration of unidentified detections, is available in Jiménez et al. (2021). In low‐density contexts, such as the peripheral regions of a species' distribution, it is expected that detection rates will be low. Under these conditions, the exclusion of photographic records due to the inability to identify individuals—often caused by partial views or poor image quality—can result in substantial data loss. This loss may reduce the accuracy and overall reliability of density estimates. The random thinning SCR model addresses this issue by incorporating unidentified detections through a probabilistic thinning process, thereby maximizing the use of available data.

We implemented a Bayesian SCR model (Royle et al. 2014) using NIMBLE (de Valpine et al. 2017, 2024) and R (R Core Team 2025), under the closed population assumption. To assess this assumption, we used the *closure.test* function from the secr package (Efford 2024), which detects potential violations during the sampling period.

The standard SCR model assumes that individual activity centers (ACs), denoted as $s=\left(s_{x},s_{y}\right)$, for each individual i=1,2,…,N, are distributed within a defined area or state space $\left(S\right)$. Individuals are detected using an array of camera traps located within S. In this study, we assumed that distribution of ACs is described by a homogeneous point process, modeled as $s_{i}\sim \text{Uniform}\left(S\right)$. These ACs are latent variables estimated by the model based on detection events recorded at various camera traps j=1,2,…J, located at coordinates $x_{j}=\left(x_{j_{1}},x_{j_{2}}\right)$.

The standard SCR model assumes that detection frequency decreases with Euclidean distance $d_{\mathrm{ij}}$ between an individual's activity center $s_{i}$ and a camera trap location $x_{i}$. This relationship was modeled using:

$$\lambda_{\mathrm{ij}}=\lambda \left(s_{i},x_{j}\right)=\lambda_{0}\times \exp\left(-\frac{d_{\mathrm{ij}}^{2}}{2\sigma^{2}}\right)$$

where $\lambda_{0}$ is the baseline detection rate (i.e., when $d_{\mathrm{ij}}=0$) and σ is the scale parameter of the half‐normal detection function and could be considered as a descriptor of the movement patterns of the target species. Under a Bayesian approach to capture–recapture with unknown N, data augmentation can be used to estimate the number of unobserved individuals (Royle et al. 2014).

$$z_{i}\sim \text{Bern}\left(\psi \right)$$

where $z_{i}$ is a partially latent binary indicator variable that describes the membership of individual i in the population, and ψ the parameter of the M data augmentation, and the membership of individual *i* in the population:

$$y_{\mathrm{ijk}}^{\text{full}}|z_{i}\sim \text{Poisson}\left(\lambda_{\mathrm{ij}}\cdot z_{i}\right)$$

To incorporate detections without individual identification, we extended the model using rt‐SCR (Jiménez et al. 2021). The loss of individual identification occurs in capture–recapture studies due to different factors (such as blurred or partial images in camera traps). The process of assigning individual identities to samples can be conceptualized as a “random thinning” process, wherein samples lose their individual identities with probability 1−θ. Thus, random thinning process generates two data sets: one comprising identified individuals and another containing unidentified encounters. The rt‐SCR model incorporates a sub model for individual identification $y_{\mathrm{ij}}^{\mathrm{ID}}$, conditional on the true encounter frequencies $y_{\mathrm{ij}}^{\text{full}}$, assuming:

$$y_{\mathrm{ij}}^{\mathrm{ID}}\sim \text{Binomial}\left(y_{\mathrm{ij}}^{\text{full}},\theta \right)$$

The unidentified encounter frequencies $y_{\mathrm{ijk}}^{\text{noID}}$ remain latent, with $y_{\mathrm{ij}}^{n\mathrm{oID}}=y_{\mathrm{ij}}^{\text{full}}-y_{\mathrm{ij}}^{\mathrm{ID}}$. For unidentified samples, only the aggregate counts in each camera trap summed across captured individuals, defined as ${\text{nnid}}_{\mathrm{jk}}=\sum_{i=1}^{N}y_{\mathrm{ijk}}^{\text{noID}}$, can be observed. The same individual may appear in both encounter histories—identified and unidentified—within a camera trap.

The rt‐SCR model was implemented in R and NIMBLE, with a Metropolis–Hastings update for $y_{\mathrm{ij}}^{\text{full}}$ that follows the constraint $y_{\mathrm{ij}}^{\text{noID}}=y_{\mathrm{ij}}^{\text{full}}-y_{\mathrm{ij}}^{\mathrm{ID}}$.

We used the zero's trick in NIMBLE, buffering the trap array by three times the estimated value of σ (9150 m, based on an initial fit of the rt‐SCR model using the full dataset). Although density estimates are generally robust to buffer sizes beyond 3σ, this choice ensures that the model focuses on the population effectively exposed to the detection array. Beyond this buffer, individuals are assumed to have a negligible detection probability and are thus considered undetectable (Royle et al. 2014). Consequently, the model implementation restricts inference to the subset of individuals with a realistic chance of being detected, enhancing both ecological relevance and statistical efficiency. To assess differences between SCR and rt‐SCR estimates, we also performed a standard SCR analysis.

We ran three MCMC chains for 500,000 iterations each, discarding the first 100,000 as burn‐in, and thinning by 10, resulting in 120,000 posterior samples per parameter. Although sex‐specific patterns may give rise to differences in σ and $\lambda_{0}$ (Tobler and Powell 2013), we opted for a null model due to our limited sample size. We used vague priors following common practice in SCR modeling (see “*Jaguar random thinning‐SCR.R*” code in Zenodo repository). These priors are routinely employed in SCR to allow the data to drive parameter estimation without imposing strong prior structure. These choices ensure that posterior estimates are primarily informed by the data while maintaining computational stability. Convergence was assessed visually and using Gelman‐Rubin diagnostics $\hat{R}$ < 1.1 (Brooks and Gelman 1998). For point estimates, we reported posterior means, except for population size, where we used posterior medians due to skewness. Uncertainty was expressed using 95% Bayesian credible intervals (CRIs). For the GoF test we generated simulated detection data for all *M* individuals in rows and *J* columns for the detectors using the Markov chain Monte Carlo (MCMC) process. We plotted the observed and posterior predictions for each statistic, and we calculated the Bayesian *p* value (used to measure the dissimilarity between observed data and model‐predicted data). The GoF was evaluated based on three statistics: total detections, number of detected individuals, and number of visited camera traps (Jiménez et al. 2022). Data and *R* + NIMBLE code used in the analysis are available in the Zenodo repository: https://doi.org/10.5281/zenodo.16943255.

## 3 Results

Of the 92 jaguar detections from 3779 trap‐days across 52 paired camera stations, 66 occurred in El Cielo and 26 in Sierra de Tamalave. Among the 17 identified individuals, 14 were detected exclusively in El Cielo, 3 in Sierra de Tamalave, as depicted using the *spiderplot* function of the scrbook package (Royle et al. 2020) (Figure S2). No individuals were recorded in both areas. We identified individuals from 81 events, of which nine were adult females and three were adult males, whereas five individuals could not be sexed due to limited photographic angles. For the remaining events (*n* = 11), identification was not possible because the low‐quality images did not allow for rosette pattern observation. Two resident male jaguars and three females accounted for the majority of detections (*n* = 36 and *n* = 42, respectively). Notably, a female accompanied by two cubs was the most frequently detected female during this study (*n* = 17) (Figure S3). One significant finding of the study was the detection of three breeding females, two of which were observed accompanied by a single cub each, whereas the third was accompanied by two cubs. A pregnant female was also identified. The mean of the maximum observed distances between cameras for individuals detected at multiple sites was 6452 m (range: 2832–11,402 m).

### 3.1 Population Density

**FIGURE 3:**
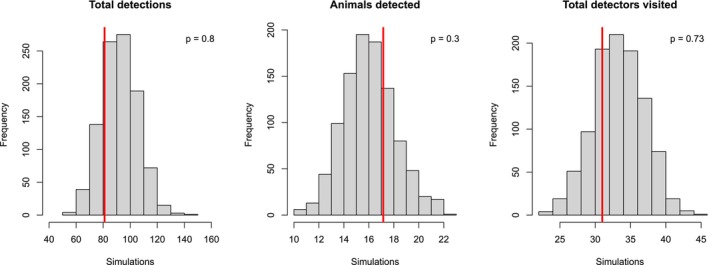
Histogram plots of observed values (red vertical lines) and posterior predictive values for the random thinning spatial capture–recapture model. In each plot, the Bayesian *p* value is shown (top right).

**FIGURE 4:**
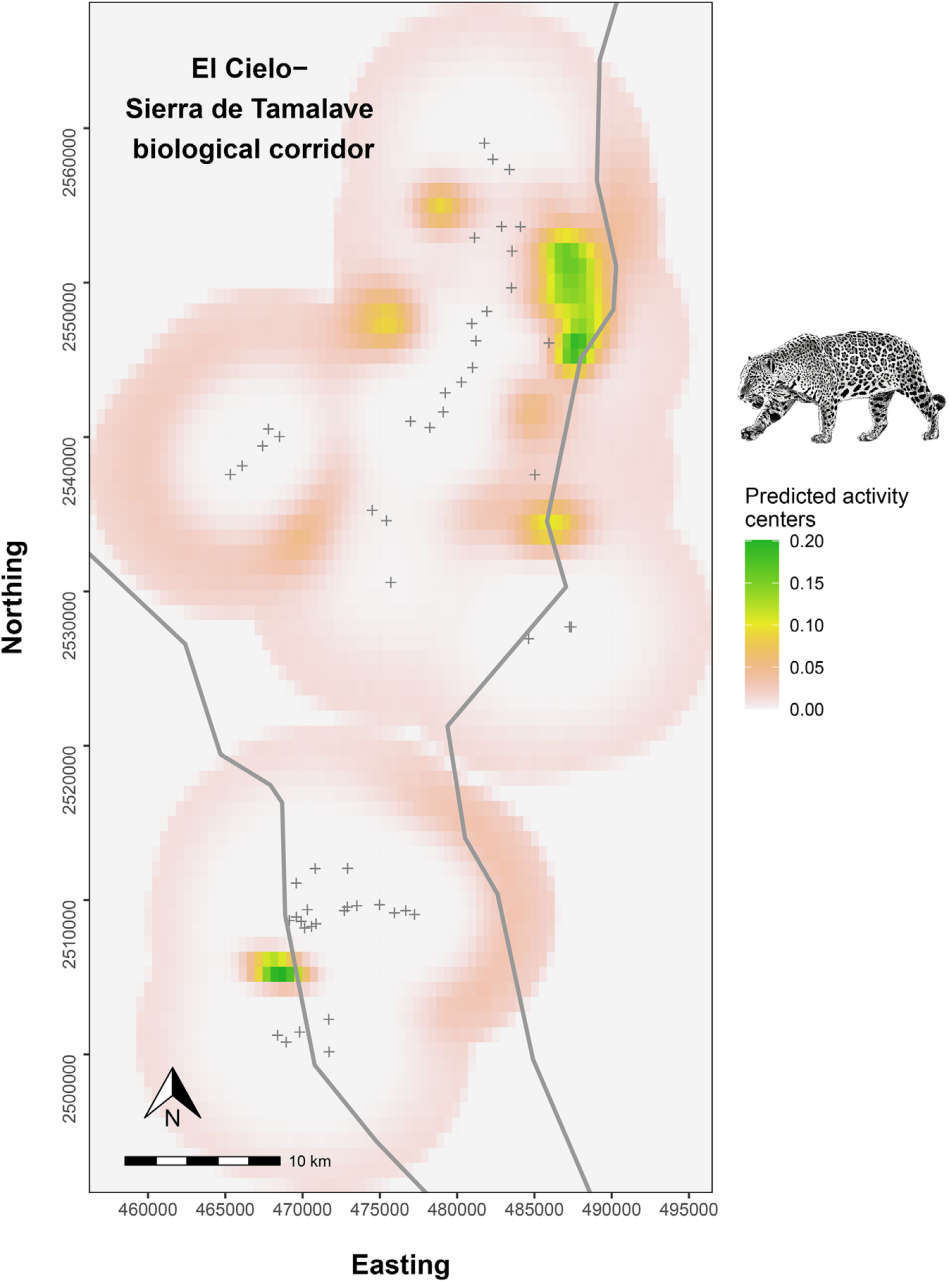
Predicted distribution of activity center location surface of jaguar in El Cielo–Sierra de Tamalave biological corridor (Tamaulipas, Mexico) estimated using random thinning spatial capture–recapture modeling, based on the spatial detection histories in camera traps (gray crosses indicate trap locations). Jaguar illustration credit: Jesús Rodriguez Osorio.

Closure test indicated no violation of the closure assumption (*Z* = −1.19), *p* = 0.118 (> 0.05). In light of these results, the closure assumption appears reasonable for the 91‐day sampling period used in our dataset. The posterior estimate of jaguar population size within the defined state space—delimited by a buffer of 3*σ* around the camera trap array (1941 km^2^)—was 25 (18–33) individuals. The density estimated was 1.29 (0.93–1.70) individuals/100 km^2^, and a coefficient of variation (CV) of 0.17. Baseline detection rate ($\lambda_{0}$) was 0.047 (0.02–0.08) and σ, 3.05 (2.55–3.53) km. Hence, using the relationship from Royle et al. (2011), the jaguar home range calculated from σ was 17,604 (12,547–24,630) ha. The identification rate in our detections was 0.87 (0.81–0.94). The GoF was adequate for the three statistics studied; Bayesian *p* values for total detections, detected individuals, and total visited camera traps were 0.80, 0.30 and 0.73, respectively (Figure 3). Comparative metrics between SCR and rt‐SCR for abundance (*N*), spatial scale (σ), and density (*D*) are presented in Table S1. Overall, the model provided highly precise estimates and an adequate GoF, indicating that additional complexity was unnecessary. The prediction of activity centers (Figure 4) shows a greater presence of the species in the northern part of the study area.

## 4 Discussion

The El Cielo–Sierra de Tamalave biological corridor constitutes one of the most important connections for jaguar conservation in northeastern Mexico (Steinberg et al. 2014). However, there is a notable lack of robust ecological studies on jaguar populations in this region. Our study provides the first robust estimate of jaguar density for the El Cielo–Sierra de Tamalave corridor using the rt‐SCR. The estimated density (1.29 jaguars per 100 km^2^) is supported by model fit diagnostics. Despite placing cameras in locations favored by direct or indirect evidence of medium‐ to large‐sized carnivores and their prey (tracks, scats, scrapes, trails), detections remained scarce—an expected outcome in low‐abundance contexts.

Beyond density, our detections offer insight into the observed individual structure of the local population: we documented a resident subpopulation comprising nine adult females, three adult males, five unsexed individuals, and four cubs, including three breeding females (two accompanied by one cub and one accompanied by two cubs) and a pregnant female. These observations provide direct evidence of reproduction within the corridor, supporting the view that El Cielo–Sierra de Tamalave functions not only as a movement conduit but also as reproductive habitat for jaguars at the northeastern edge of their distribution. These demographic observations rely exclusively on the individuals detected during the survey, since the small sample size prevented a formal SCR‐based analysis of age structure, sex ratio, or other demographic attributes. Such analyses are planned for subsequent research.

Methodologically, we adopted the rt‐SCR approach to maximize the available sample size and minimize bias when recaptures are scarce—a common challenge for elusive species, peripheral populations, and rugged landscapes (Jiménez et al. 2021). However, two limitations warrant attention. First, the low number of detections precluded modeling sex‐specific differences (Tobler and Powell 2013). Second, steep slopes, ravines, and restricted access limited systematic sampling and necessitated targeted camera placement, reducing spatial resolution for estimating activity centers and inferring space use (see Figure 4). In this specific application, the rt‐SCR model yielded only a modest improvement in precision relative to the standard SCR model (Table S1). Although the reduction in the coefficient of variation (CV) for abundance (*N*) is small (−2.59%), the decrease in the CV for σ is more pronounced (−5.69%). Because σ defines the spatial scale of detection, it directly calibrates the state space. As density (*D*) derives from abundance and area, a better‐calibrated state space reduces bias and yields more reliable density estimates, even if the observed improvement in *D*'s CV (−2.63%) remains modest.

The northernmost populations of the jaguar's distribution in Mexico (e.g., Tamaulipas, Sonora, Nuevo León, Sinaloa) remain underrepresented in systematic jaguar studies, which hinders the design of tailored conservation strategies for range‐margin populations (Ceballos et al. 2021; Charre‐Medellín et al. 2021; Amador‐Alcalá et al. 2024). By delivering a spatially explicit density estimate for the El Cielo–Sierra de Tamalave corridor, our results help fill a critical knowledge gap and provide a foundation for evidence‐based management in a key connectivity zone linking central Mexican populations with northern ones along the Gulf slope (Carrera‐Treviño et al. 2016; Gómez et al. 2018) (Table 1).

**TABLE 1:** Jaguar studies in Mexico reporting density estimates (ind./100 km^a^) based on camera trap surveys (*n* = 31).

Region	State	Monitoring site	Number of stations	Model	Density	Sampling year	Distribution limit
Northern Mexico	Sonora	Northern Sonora^b^	164	SPIM‐SCR	1.54–4.61	2009	●
		Jaguar del Norte Reserve^a^, ^c^	56	SCR	0.21–3.04	2014	●
			56	CR‐(MMDM)	0.3–1.44	2014	
			25–111	Open CR‐(MMDM)	1.07	2009–2010	●
		Nácori Chico^d^	66	CR‐(½MMDM)	1.1	2005	●
	Tamaulipas	El Cielo^e^	22	CR‐(MMDM)	5.9	2013–2014	●
		This study	52	rt‐SCR	1.29	2023	●
	Sinaloa	PNA Meseta de Cacaxtla^f^	7–10–7	CR‐Telemetry	1.59	2007–2008	○
Central México	San Luis Potosí	Biosphere Reserve Sierra del Abra Tanchipa^g^	51–24	CR‐(½MMDM)	2.29	2010–2011‐2012	○
	Querétaro	Sierra Gorda^h^, ^i^	—	CR‐(MMDM)	0.75	2006–2007	○
			60	CR‐(MMDM)	0.9	2014	○
	Nayarit	Central Pacific Jaguar Conservation Unit^j^	25	SCR	1.62–1.95	2019–2020	○
		Marismas nacionales^k^, ^l^	30	CR‐(½MMDM)	2.5–5.9	2011	○
			52	SCR	2.04	2010	○
	Jalisco	Chamela‐Cuixmala^m^	29	CR‐(½MMDM)	5.3	2008	○
	Michoacán	San José de Los Pinos^n^	27	CR‐(½MMDM)	1.46–1.63	2014–2015	○
			27	CR‐(MMDM)	0.68–0.79	2014–2015	
			27	SCR	0.73–0.76	2014–2015	
Southern Mexico	Oaxaca	Chinantla^o^	38	SCR	1.15–1.16	2015	○
	Chiapas	Montes Azules^p^	16–33–42	CR‐(MMDM)	1.7–3	2007–2008	○
			16–33–42	CR‐(½MMDM)	2.6–4.6		
	Campeche	Pantanos de Centla^q^	103	SCR	1.9	2016	○
		Gran Calakmul Región^r^, ^s^, ^t^	27	—	4.5	—	○
			69	SCR	1.68–2.63	2018–2019‐2022	○
			38	SCR	1.03	2021–2022	○
	Quintana Roo	El Edén^u^	27–22–24	SCR	1.10–2.23	2008–2010–2011‐2012	○
			27–22–24	CR‐(MMDM)	1.58–2.63		
			27–22–24	CR‐(½MMDM)	3.08–5		
		Ría Lagartos^r^	27	No reported	2.6	—	○
		Yum Balam^r^	27	No reported	7.4	—	○
	Yucatán	Ría Lagartos^v^	18–27	CR‐(½MMDM)	3–6	2004–2005‐2006	○

*Note:* Non‐spatial capture–recapture based on mean maximum distance moved (CR‐MMDM or ½MMDM); random thinning spatial capture–recapture (rt‐SCR); spatial capture–recapture (SCR); spatial partial identity spatial capture–recapture (SPIM‐SCR). Black points (●) represent studies conducted at the jaguar's distributional edge, whereas white points (○) indicate studies carried out in the species' core distribution zones within Mexico.

^a^ Amador‐Alcalá et al. (2024).

^b^ Greenspan et al. (2020).

^c^ Gutiérrez‐González et al. (2012).

^d^ Rosas‐Rosas and Bender (2012).

^e^ Carrera‐Treviño et al. (2016).

^f^ Coronel‐Arellano et al. (2017).

^g^ Hernández‐SaintMartín (2014).

^h^ Coronel‐Arellano et al. (2008).

^i^ Anaya‐Zamora et al. (2015).

^j^ Murphy and Luja (2025).

^k^ RBMN (2013).

^l^ Figel et al. (2016).

^m^ Nuñez (2011).

^n^ Charre‐Medellín et al. (2021).

^o^ Lavariega et al. (2020).

^p^ de la Torre and Medellín (2011).

^q^ Hidalgo‐Mihart et al. (2019).

^r^ Ceballos et al. (2016).

^s^ Hidalgo‐Mihart et al. (2025).

^t^ Contreras‐Moreno et al. (2025).

^u^ Ávila‐Nájera et al. (2015).

^v^ Faller et al. (2007).

Comparisons of density across Mexico must account for methodological differences. Non‐spatial CR estimates can be biased and often overestimate density; thus, comparisons should be restricted to spatially explicit models (Sutherland et al. 2016; Tobler and Powell 2013; Murphy and Luja 2025). In peripheral regions, reported densities commonly range around one to two individuals per 100 km^2^—for example, Meseta de Cacaxtla (1.59; Coronel‐Arellano et al. 2017), Northern Jaguar Reserve (1.07 and 1.44; Gutiérrez‐González et al. 2012; Amador‐Alcalá et al. 2024), northern Sonora (1.1; Rosas‐Rosas and Bender 2012), pine‐oak forests of Sonora (1.54; Greenspan et al. 2020)—which is consistent with our El Cielo–Sierra de Tamalave estimate (Table 1). Densities are generally higher in the southern region of Mexico in core areas, such as the Yucatán Peninsula—Calakmul (4.5; Ceballos et al. 2016), El Edén (5.0; Ávila‐Nájera et al. 2015), Río Lagartos (6.0; Faller et al. 2007), and Chamela–Cuixmala in western Mexico (5.4; Nuñez 2011)—yet recent, spatially explicit updates in Calakmul (1.03–2.63; Hidalgo‐Mihart et al. 2025; Contreras‐Moreno et al. 2025) underscore how landscape context, prey availability, and methods jointly shape estimates (Tobler and Powell 2013; Jiménez et al. 2021).

Anthropogenic pressures around El Cielo–Sierra de Tamalave—agricultural expansion (sugarcane, citrus), induced pastures for cattle, new and expanding settlements, and road infrastructure—are compromising structural connectivity, especially in low‐slope areas and rolling hills that cross and border the mountainous region (Gómez et al. 2018). Such pressures mirror wider patterns across the species' range (Luja et al. 2022), with implications for prey communities (Ochoa‐Espinoza et al. 2023) and potential retaliatory hunting in livestock areas (Lavariega et al. 2020; Amador‐Alcalá et al. 2024). In this context, peripheral corridors like El Cielo–Sierra de Tamalave may contribute disproportionately to population resilience by maintaining genetic variability and enabling gene flow to core zones (Ceballos et al. 2016; Sanderson et al. 2022), provided their connectivity and habitat quality are conserved.

Our results support targeted actions to maintain and enhance ecological connectivity in the El Cielo–Sierra de Tamalave corridor: habitat restoration in degraded low‐slope areas, maintenance of forest cover in mountainous sectors, and mitigation of human–wildlife conflict in livestock landscapes (Gómez et al. 2018). Given that jaguar populations can persist at low densities even in core regions (Ceballos et al. 2016; Hidalgo‐Mihart et al. 2025), long‐term monitoring with spatially explicit methods should be prioritized to refine estimates, detect demographic trends, and inform adaptive management. Sustained collaboration among government agencies, civil associations, and local communities remains essential for generating the information needed to protect range‐margin populations.

## 5 Conclusion

By applying rt‐SCR in a challenging, peripheral landscape, we provide a spatially explicit density estimate for El Cielo–Sierra de Tamalave that aligns with other range‐margin values. In addition, field detections of females with offspring document active reproduction within the corridor, complementing the model‐based inference. Although jaguar density is low, the clustering of activity centers together with evidence of breeding indicates that the corridor serves a dual role: it offers refuge for resident individuals and facilitates connectivity between larger populations. Protecting and managing El Cielo–Sierra de Tamalave to sustain connectivity, while addressing anthropogenic pressures, will be critical for supporting jaguar movement, reproduction, and long‐term viability at the northeastern limit of the species' distribution.

## Author Contributions

**Zavdiel A. Manuel‐de la Rosa:** conceptualization (equal), data curation (equal), formal analysis (equal), writing – original draft (lead), writing – review and editing (equal). **Leroy Soria‐Díaz:** conceptualization (equal), methodology (equal), supervision (lead), writing – original draft (equal), writing – review and editing (equal). **Carlos Barriga‐Vallejo:** conceptualization (equal), methodology (equal), project administration (lead), writing – original draft (equal), writing – review and editing (equal). **Gabriela R. Mendoza‐Gutiérrez:** data curation (equal), investigation (equal), writing – review and editing (equal). **Nayeli Martínez‐González:** data curation (equal), investigation (equal), writing – review and editing (equal). **Claudia C. Astudillo‐Sánchez:** data curation (equal), investigation (equal), writing – review and editing (equal). **José Jiménez:** conceptualization (equal), formal analysis (equal), software (lead), writing – original draft (equal), writing – review and editing (equal).

## Funding

This work was supported by the US Fish and Wildlife Service (FWS‐IA2022001369).

## Conflicts of Interest

The authors declare no conflicts of interest.

## Supporting information


**Figure S1:** Camera traps operation plot. Light and dark blue are active and inactive detectors, respectively.
**Figure S2:** This figure depicts individual detection patterns and spatial recapture dynamics across the camera trap array. Black lines denote spatial recapture trajectories between distinct trap locations, whereas colored points represent the centroid of capture events per individual, calculated from all recorded detections. The diameter of the light green circles is proportional to the cumulative number of capture events—encompassing both identified (ID) and non‐identified (non‐ID) occurrences—registered at each camera trap site.
**Figure S3:** Evidence of jaguar cubs within the El Cielo–Sierra de Tamalave biological corridor. Image credit: Carlos Barriga‐Vallejo/Pronatura Noreste.
**Table S1:** Comparative summary of posterior estimates from standard spatial capture–recapture (SCR) models and random thinning SCR (rt‐SCR) models applied to the jaguar ( *Panthera onca* ) population in El Cielo–Sierra de Tamaulipas, northeastern Mexico. Parameters: *D* = density (individuals per 100 km^2^); *N* = estimated number of individuals within the state space; $\lambda_{0}$= baseline detection rate; σ = scale parameter of the half‐normal detection function, indicative of movement (in kilometers).

## Data Availability

The data and code (Manuel‐de la Rosa et al. 2025) are available in the Zenodo repository at https://doi.org/10.5281/zenodo.16943255.
