# Supplementary material for: Jaguar Density at the Northeastern Limit of Its Distribution in Mexico
Source: Ecol Evol. 2026 Feb 3;16(2):e72932. doi: 10.1002/ece3.72932 (PMC12865508; doi:10.1002/ece3.72932)
Supplement: Supplementary file 1 — Figure S1: Camera traps operation plot. Light and dark blue are active and inactive detectors, respectively. Figure S2: This figure depicts individual detection patterns and spatial recapture dynamics across the camera trap array. Black lines denote spatial recapture trajectories between distinct trap locations, whereas colored points represent the centroid of capture events per individual, calculated from all recorded detections. The diameter of the light green circles is proportional to the cumulative number of capture events—encompassing both identified (ID) and non‐identified (non‐ID) occurrences—registered at each camera trap site. Figure S3: Evidence of jaguar cubs within the El Cielo–Sierra de Tamalave biological corridor. Image credit: Carlos Barriga‐Vallejo/Pronatura Noreste. Table S1: Comparative summary of posterior estimates from standard spatial capture–recapture (SCR) models and random thinning SCR (rt‐SCR) models applied to the jaguar ( Panthera onca ) population in El Cielo–Sierra de Tamaulipas, northeastern Mexico. Parameters: D = density (individuals per 100 km2); N = estimated number of individuals within the state space; λ0= baseline detection rate; σ = scale parameter of the half‐normal detection function, indicative of movement (in kilometers). [file ECE3-16-e72932-s001.docx]

**Jaguar density at the northeastern limit of its distribution in México**

**Appendix S1**

Zavdiel A. Manuel-de la Rosa^1^, Leroy Soria-Díaz^1, 2^, Carlos Barriga-Vallejo^1, 3^, Gabriela R. Mendoza-Gutiérrez^1^, Nayeli Martínez-González^1^, Claudia C. Astudillo-Sánchez^4^, José Jiménez^5^

^1^ Instituto de Ecología Aplicada, Universidad Autónoma de Tamaulipas, Av. División del Golfo, Ciudad Victoria C.P. 87019, Tamaulipas, México.

^2^ Facultad de Medicina Veterinaria y Zootecnia “Dr. Norberto Treviño Zapata”, Universidad Autónoma de Tamaulipas, Carretera Victoria-Mante km 5, Santa Librada, Ciudad Victoria C.P. 87274, Tamaulipas, México.

^3^ Pronatura Noreste, Loma Grande 2623, Col. Loma Larga, Monterrey C.P. 64710, Nuevo León, México.

^4^ Facultad de Ingeniería y Ciencias, Universidad Autónoma de Tamaulipas, Centro Universitario Victoria, Ciudad Victoria C.P. 87149, Tamaulipas, México.

^5^ Instituto de Investigación en Recursos Cinegéticos (IREC, CSIC-UCLM-JCCM), Ronda de Toledo, 12, C.P. 13005, Ciudad Real, España.

Figure S1. Camera-traps operation plot. Light and dark blue are active and inactive detectors, respectively.

Figure S2. This figure depicts individual detection patterns and spatial recapture dynamics across the camera trap array. Black lines denote spatial recapture trajectories between distinct trap locations, while coloured points represent the centroid of capture events per individual, calculated from all recorded detections. The diameter of the light green circles is proportional to the cumulative number of capture events—encompassing both identified (ID) and non-identified (non-ID) occurrences—registered at each camera trap site.


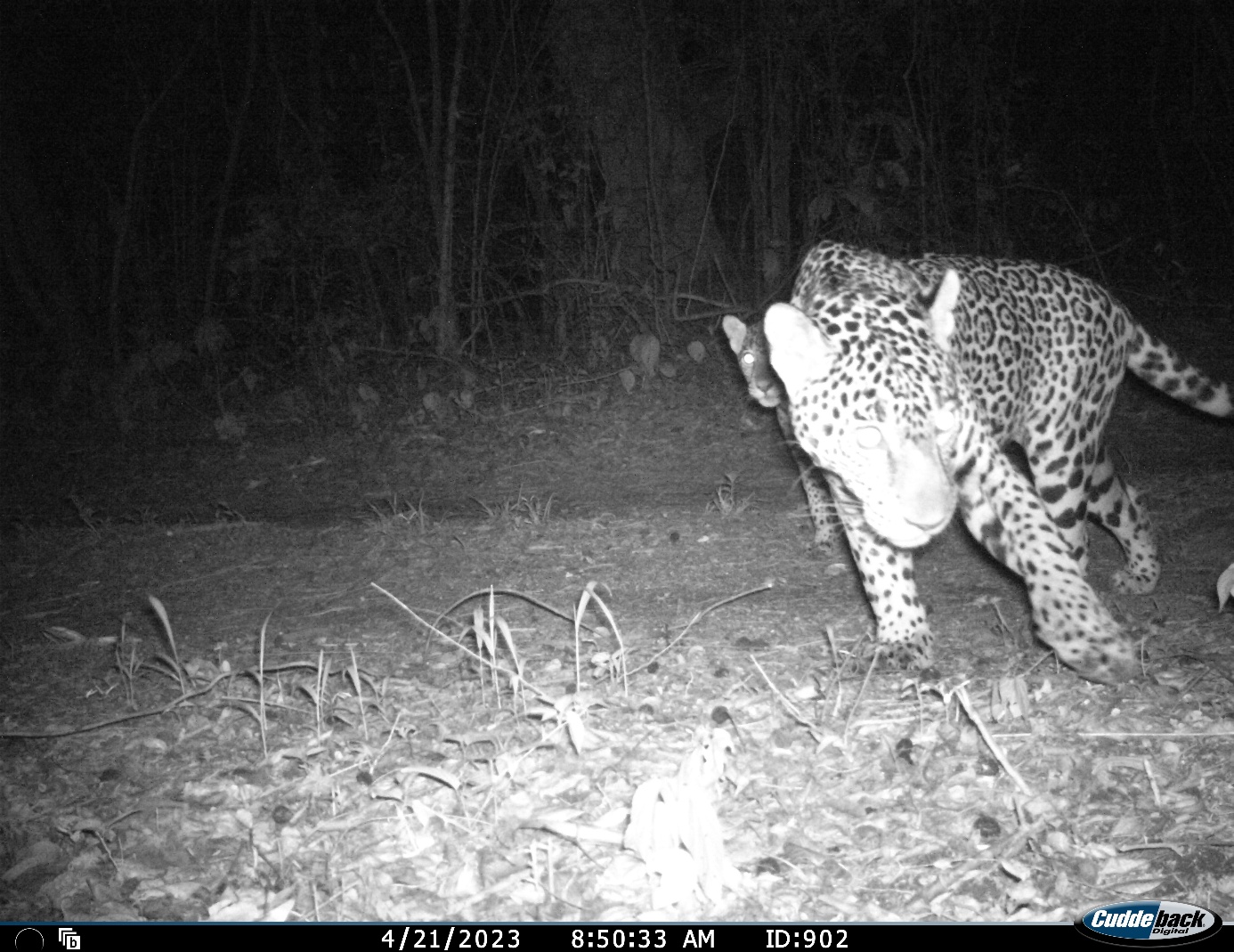


Figure S3. Evidence of jaguar cubs within the El Cielo–Sierra de Tamalave biological corridor. Image credit: Carlos Barriga-Vallejo / Pronatura Noreste.

Table S1. Comparative summary of posterior estimates from standard Spatial Capture–Recapture (SCR) models and Random Thinning SCR (rt-SCR) models applied to the jaguar (*Panthera onca*) population in El Cielo–Sierra de Tamaulipas, northeastern Mexico. Parameters: D = density (individuals per 100 km²); N = estimated number of individuals within the state space; $\lambda_{0}$ = baseline detection rate; $\sigma$ = scale parameter of the half-normal detection function, indicative of movement (in kilometers).

|  | **SCR** | | | | **rt-SCR** | | | |
| --- | --- | --- | --- | --- | --- | --- | --- | --- |
| Parameter | **Mean** | **SD** | **lower95** | **upper95** | **Mean** | **SD** | **lower95** | **upper95** |
| D | 1.313 | 0.227 | 0.979 | 1.803 | 1.301 | 0.219 | 0.927 | 1.700 |
| N | 25.481 | 4.413 | 18.000 | 34.000 | 25.245 | 4.259 | 18.000 | 33.000 |
| $\lambda_{0}$ | 0.038 | 0.012 | 0.019 | 0.062 | 0.047 | 0.014 | 0.024 | 0.076 |
| $\sigma$ | 3.135 | 0.289 | 2.592 | 3.706 | 3.048 | 0.265 | 2.547 | 3.572 |
